# Birds and beans: Comparing avian richness and endemism in *arabica* and *robusta* agroforests in India’s Western Ghats

**DOI:** 10.1038/s41598-018-21401-1

**Published:** 2018-02-16

**Authors:** Charlotte H. Chang, Krithi K. Karanth, Paul Robbins

**Affiliations:** 1 Department of Ecology and Evolutionary Biology, Princeton University, Princeton, NJ 08544 USA; 2 Wildlife Conservation Society, 2300 Bronx Blvd, New York, USA; 3 Centre for Wildlife Studies, 551, 7th Main Road Rajiv Gandhi Nagar, 2nd Phase, Kodigehalli, Bengaluru, 560097 India; 4 Duke University, Durham, North Carolina USA; 5 Nelson Institute, University of Wisconsin-Madison, Madison, Wisconsin USA

## Abstract

Coffee is a major tropical commodity crop that can provide supplementary habitat for native wildlife. In Asia, coffee production is an increasingly important driver of landscape transformation and shifts between different coffee species is a major dimension of agroforestry trends. Yet few studies have compared the ecological impacts of conversion between different coffee species. We evaluated whether or not the two species of coffee grown globally—*Coffea arabica* and *C. canephora* (denoted “*robusta*”)—had equivalent avian conservation value in the Western Ghats, India, where *robusta* production has become increasingly dominant. We found that habitat specialist and functional guild diversity was higher in *arabica*, and that *arabica* was more profitable. However, *robusta* farms generally supported the same or slightly higher abundances of habitat specialists and functional guilds, largely due to dense canopy and landscape-level forest cover. Farming practices, chiefly pesticide use, may affect the suitability of coffee agroforests as habitat for avian specialists, and at present, *robusta* farmers tended to use less pesticide. Given future projections for *arabica* to *robusta* conversion in tropical Asia, our study indicates that certification efforts should prioritize maintaining native canopy shade trees and forest cover to ensure that coffee landscapes can continue providing biodiversity benefits.

## Introduction

Coffee (*Coffea* spp.) is one of the most valuable and widely planted tropical commodity crops with two major species in production: *C. arabica* (henceforth, *arabica*), constituting about 60% of global production, and *C. canephora* (*robusta*), around 40% of global production^1^. Coffee production systems range from shaded, low-intensity farming where coffee trees are interspersed with native forest, more typical of *arabica* production, to high-intensity full-sun monoculture characteristic of *robusta* agroforests. Shade-grown coffee retains biodiversity at higher levels than more intensely farmed, full-sun or monoculture systems^2–5^.

Shade grown coffee has declined precipitously in the past twenty years^6,7^, largely due to greater *robusta* production, particularly in Asia^8–11^. Price equalization between the two species and declining *arabica* productivity in the face of climate change may further accelerate conversion to *robusta* across tropical Asia^12,13^. Understanding habitat specialist responses to *arabica* versus *robusta* is critical as production may shift away from *arabica* toward *robusta* in many parts of the tropics.

Demand for coffee is rising much more rapidly in Asia (3.7%) than the global average (1.3%)^14^. While coffee production has declined in the Neotropics and Afrotropics, in South and Southeast Asia, it has increased by more than 100%^7^. Currently, India is the world’s sixth largest coffee producer and coffee acreage in India has increased by 150% from 1990 to 2015^15,16^. The majority of this expansion occurred in a global biodiversity hotspot, the Western Ghats^17–19^.

Across the Western Ghats, coffee agroforest area is slightly more than a quarter of the land area that is formally protected^16^; whether coffee can serve as buffer habitat for wildlife will be critically important to conservation outcomes^19,20^. From 1950 to 2015, the planted area of *robusta* grew by 840% while *arabica* acreage increased by 327% in India^16^. Previous research posited that *robusta* expansion could be ecologically detrimental as is often grown in more open conditions where farmers fell older trees and lop more branches to open up the canopy^21^.

In this article, we analyze avian habitat specialist trends in *arabica* and *robusta* farms and integrate household interviews to explore what policy levers may be most powerful in securing a biodiversity-friendly future for coffee production lands. Despite coffee’s importance as a driver of landscape transformation, and the unclear impacts of *robusta* supplanting *arabica*, there have been limited studies comparing these two agroforests from a biodiversity perspective^22^. The majority of existing assessments have often not distinguished between coffee species, and have instead compared coffee against other crops, sacred groves, and native forests^19,23–28^.

## Results

### *Arabica* and *robusta* production practices and trends

Across the entire household survey dataset (n = 344), 213 households planted *arabica* and 236 planted *robusta*. Of the *arabica* planters, 50.2% solely planted *arabica*; for *robusta* planters, 55.1% solely planted *robusta*; 106 households grew both varieties of coffee. In 2003, 215 of the respondents produced *robusta* and 196 farmed *arabica*. Compared to this baseline, the net change in the number of farmers planting each species was a 9.3% increase for *robusta* and 7.7% increase for *arabica*.

**Figure 1:**
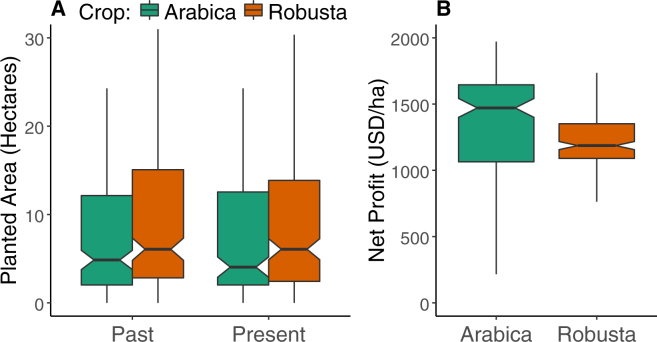
(**A**) Changes in planted area for *arabica* and *robusta* among the surveyed households. Notched boxplots of planted area a decade ago and during the studied period for both crops. (**B**) The net profits for each crop (in 2013 USD$) per unit hectare. After subtracting the cost of inputs per hectare, *arabica* was more profitable than *robusta* on average.

The average planted area of *arabica* was 14.3 ± 0.1 hectares (*n* = 213, median: 4.9 ha, maximum: 242.8 ha) and 11.6 ± 0.1 hectares for *robusta* (*n* = 236, median: 6.1 ha, maximum: 121.4 ha). At the time of the study, the mean planted areas for each crop were not significantly different (W = 22510, p_adj_ = 0.2, Fig. 1A). On the other hand, the mean acreage of *robusta* increased significantly over the past decade (V = 977, p_adj_ = 3.7 × 10^−6^, Fig. 1A).

Both *arabica* and *robusta* stands had fairly closed canopies with a median canopy density score of 94.6% (mean: 94.6%, range: [79.7, 99.7]%) for *arabica* and 79.2% for *robusta* (mean: 77.2%; [50.4, 99.8]%). Moreover, all coffee agroforests tended to be situated in regions with dense forest cover with a median tree cover of 92.1% for *arabica* within a 2 km buffer around each farm (mean: 88.8%, [67.5, 99]%) and 88.9% for *robusta* (mean: 86.3%, [55.3, 98.7]%).

Farmers employed a variable usage rate for five surveyed inputs; for *arabica*, the majority of farmers used pesticides as well as organic and conventional fertilizer, while for *robusta*, the majority of farmers used conventional fertilizer, and less than half of the farmers used the other four inputs (Table 1). The cost per hectare spent on each type of input was wide-ranging for both coffee species (Table 1). *Arabica* was significantly more profitable per unit area than *robusta* (*arabica*: $1555.98 ± 2.7 versus *robusta*: $1439.53 ± 2.2; W = 26407, p_adj_ = 0.002; Fig. 1B).

**Table 1:** Per hectare costs (in 2013 USD) for five major inputs across the two coffee species.

Input	Arabica				Robusta			
	User %	Pay %	Mean (SE)	Range	User %	Pay %	Mean (SE)	Range
Conventional Fertilizer	86.4	30.5	16.4 (0.25)	[0, 368.7]	82.2	70.3	46.2 (0.46)	[0, 1075]
Herbicide	18.3	14.1	4.7 (0.14)	[0, 294.9]	5.9	5.1	0.6 (0.01)	[0, 36.9]
Irrigation	17.4	17	16.3 (0.25)	[0, 123.7]	36.4	36	11.7 (0.13)	[0, 309.7]
Organic Fertilizer	48.8	31	15.9 (0.24)	[0, 368.7]	40.3	24.2	9.5 (0.15)	[0, 327.7]
Pesticide	75.6	69	31.8 (0.28)	[0, 435.7]	16.9	16.1	3.2 (0.04)	[0, 81.9]

### Avian richness patterns across the two coffee species

Across the sampled agroforests, the number of forest-dependent species ranged from 22–63 with a total of 79 forest-dependent species recorded in the full dataset. Fourteen endemic species were observed with anywhere from two to seven species found in the individual agroforests. Three IUCN Red-Listed species were observed in the course of study: Alexandrine Parakeet (*Psittacula eupatria*, near-threatened), Grey-headed Bulbul (*Pycnonotus priocephalus*, near-threatened), and Nilgiri Wood-pigeon (*Columba elphinstonii*, vulnerable). Only three agroforests did not have a single Red-listed species present Twenty-six frugivorous, 54 insectivorous, and 26 omnivorous species were recorded.

*Arabica* supported more speciose assemblages of forest-dependent, endemic, frugivorous, insectivorous, and omnivorous birds (Table 2). There were nearly twice as many endemic bird species in *arabica* compared to *robusta* (Table 2, *n*_*a,j1*_ = 19 ± 2.2, *n*_*r,j1*_ = 11 ± 0). For IUCN Red-Listed species richness, *arabica* and *robusta* agroforests had indistinguishable asymptotic richness, though this analysis was limited by the small number of threatened species (Table 2).

**Table 2:** Asymptotic richness for avian groups categorized by habitat specialization or threat across *arabica* and *robusta* stands.

Category	Observed		Chao		First-order jackknife	
	*Arabica*	*Robusta*	*Arabica*	*Robusta*	*Arabica*	*Robusta*
Forest-dependent	74	66	88 (10.3)	66.3 (0.8)	86.9 (3.9)	68 (1.4)
Endemic	14	11	20.2 (7.5)	11 (0)	19 (2.2)	11 (0)
IUCN Red-Listed	3	3	3 (0)	3 (0)	3 (0)	3 (0)
Frugivore	22	22	28.2 (7.5)	22.7 (1.3)	27 (2.2)	24 (1.4)
Insectivore	51	48	68.9 (23.5)	50 (3.7)	57 (2.4)	50 (1.4)
Omnivore	22	20	30.9 (10.1)	22.2 (3.4)	28 (2.4)	23 (1.7)

Observed: Observed richness in each habitat type across all point count stations. Chao (standard error) corresponds to the Chao 2 estimator of asymptotic richness. The first-order jackknife (standard error) is another asymptotic richness estimator.

The five most commonly observed forest-dependent, endemic, and functional guild species were similar across *robusta* and *arabica* (Appendix 1, Table A1). However, the forest-dependent Malabar grey hornbill (*Ocyceros griseus*), frugivorous Plum-headed parakeet (*Psittacula cyanocephala*), and insectivorous Oriental magpie-robin (*Copsychus saularis*) exhibited different patterns in commonness across the two coffee species (Appendix 1, Table A1).

### Habitat specialist species accumulation and community composition

**Figure 2:**
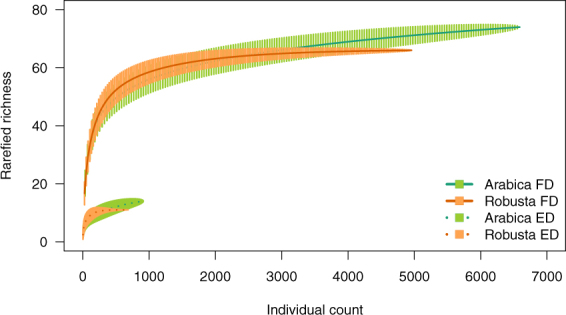
Individual-based rarefaction for *arabica* and *robusta* agroforests for forest-dependent (FD) and endemic species richness (ED).

A sufficient number of endemic and forest-dependent species were observed to conduct individual-based rarefaction. The rarefaction results indicated that the initial accumulation of forest-dependent and endemic species was similar across *arabica* and *robusta*; however, the asymptotic richness of endemics and forest birds were higher in *arabica* and more individuals were observed for both these groups in *arabica* than in *robusta* (Fig. 2).

**Figure 3:**
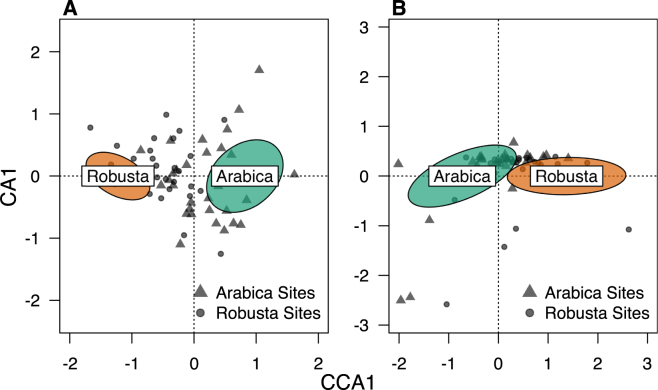
Community composition of (**A**) Forest Dependent birds and (**B**) Endemics across *arabica* and *robusta* agroforests. Ordination scores were calculated at the level of farms for each set of species using correspondence analysis. The 95% confidence ellipses for the *arabica* and *robusta* site centroids are shown in green and orange, respectively.

A permutational MANOVA demonstrated that the forest-dependent and endemic communities were significantly different between the two coffee species (F_FD_ = 3.95, *p*_*adj*_ = 0.006; F_ED_ = 3.87, *p*_*adj*_ = 0.02). For endemics and forest birds, there were no significant partitioning effects associated with environmental variables (tree richness, canopy density, and distance to protected area). Correspondence analysis (CCA) indicated that the forest-dependent and endemic species communities occupied non-overlapping ordination space (Fig. 3).

### Abundance patterns for habitat specialists, threatened birds, and foraging guilds

#### Forest dependent species

The mean detection of forest-dependent individuals was 0.09 while it was 0.14 for clusters. A range of [0.4, 48.7] forest-dependent birds per hectare were detected in *arabica* and [0.3, 28.3] in *robusta*. The range of flocks per hectare (clusters) that were observed in the two coffee species were [0.4, 19.9] flocks/ha in *arabica* and [0.4, 15] in *robusta*. There were an average of 1.54 ± 0.04 birds per flock in *arabica* and 1.46 ± 0.03 in *robusta*. We found that *robusta* supported significantly higher densities of forest-dependent flocks, but not individual birds (Table 3).

**Table 3:** Individual bird and flock densities for different avian groups in *arabica* or *robusta*.

Category	Individual Bird		Flocks	
	Arabica	Robusta	Arabica	Robusta
Forest-dependent	7.09 (0.45)	8.28 (0.49)	4.62 (0.23)*	5.68 (0.28)*
Endemics	3.02 (0.27)	3.44 (0.3)	2.17 (0.13)	2.6 (0.17)
Frugivores	4.13 (0.33)	5.19 (0.38)	2.78 (0.15)*	3.65 (0.22)*
Insectivores	2.97 (0.21)	3.08 (0.2)	1.88 (0.12)	2.07 (0.13)
Omnivores	4.13 (0.29)	4.22 (0.28)	2.54 (0.16)	2.83 (0.17)

Densities are reported in birds/ha or flocks/ha (standard error in parentheses). The asterisk (*) denotes densities that were significantly different across the coffee species.

#### Endemic species abundance

The mean detectability for endemic birds was 0.09 for individuals and 0.13 for clusters. The range of individual endemics observed per hectare was [0, 33.5] in *arabica* and [0.4, 23.3] in *robusta*. For flocks, the range of clusters per hectare was [0, 9.5] in *arabica* and [0.4, 9.3] in *robusta*. The typical flock size was 1.4 ± 0.07 endemic birds per flock in *arabica* and 1.3 ± 0.05 in *robusta*. The results did not indicate any significant difference between *arabica* and *robusta* in terms of endemic bird density (Table 3).

#### IUCN Red-Listed species

There were only 20 observations of IUCN red-listed species, preventing abundance analysis; however, a global distance analysis indicated an average detection probability of 0.27.

#### Foraging guilds

Across the three foraging guilds, detection probabilities for individual birds centered on 0.1 and was around 0.14 for flocks. Individual bird densities (birds/ha) across all three guilds ranged from [0.4, 38.8] in *arabica* and [0.4, 22.6] in *robusta*. Flock densities spanned 0.4 to 13.2 clusters per hectare in *arabica* (average flock size: 1.5 ± 0.06 birds) and 0.4–9.4 in *robusta* (flock size: 1.4 ± 0.04 birds). In general, there were no significant differences in the density of individual birds or flocks among the three foraging guilds (Table 3). However frugivore flock densities were significantly higher in *robusta*.

### Relating habitat specialist diversity and functional guild abundance to ecological and land use covariates

Using a global model containing all relevant covariates (Appendix 1, Tables A2–A6), we evaluated if there was evidence of residual spatial autocorrelation for all three guilds as well as the forest-dependent and endemic species^29^. There was no evidence of residual spatial autocorrelation for the habitat specialists or foraging guilds (Moran’s I ranged from [−0.06, 0.02] with *p ∈ *[0.16, 0.41]), consistent with the findings of Karanth *et al*.^5^.

**Figure 4:**
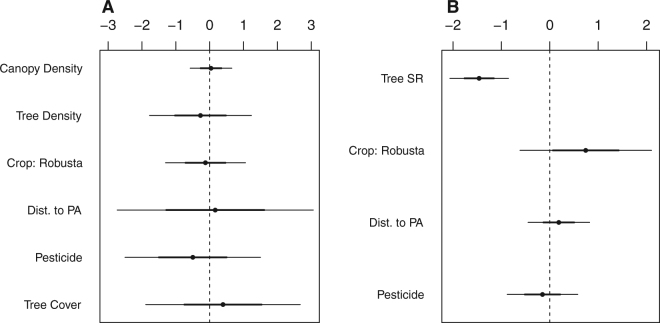
Model-averaged regression coefficients predicting (**A**) forest-dependent and (**B**) endemic species richness in *arabica* and *robusta*. The variable names are shown in column on the left. “Tree SR” represents tree species richness; “Dist. to PA” distance to protected area.

For the forest-dependent species, six of the eight candidate models garnered sufficient support for model averaging (Appendix 1, Table A2). The final ensemble model included all of the ecological covariate variables except for canopy structure and tree species richness; however, pesticide, distance to protected area, and tree cover had the highest relative variable importance scores (1, 0.82, and 0.7 respectively). Yet the confidence intervals for all variables crossed 0, indicating that none of these variables had a clear, directional effect on forest-dependent richness (Fig. 4A).

On the other hand, for the endemics, only one model was chosen under the model selection framework (weight: 0.92), as the nearest model had a ΔAICc = 5.1 (Table A2). Tree species richness, crop type, distance to protected area, and pesticide use were contained in the most-supported model. Higher tree species richness tended to decrease endemic diversity, and while there was a trend toward *robusta* predicting higher endemic diversity, this variable ultimately was not significant (Fig. 4B).

**Figure 5:**
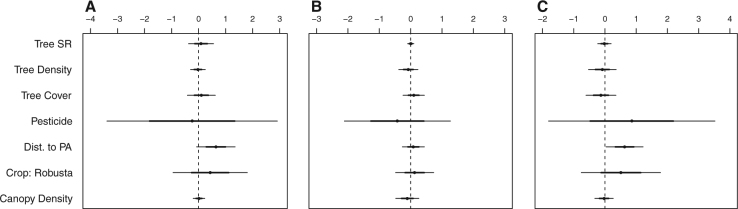
Model-averaged regression coefficients predicting (**A**) frugivore, (**B**) insectivore, and (**C**) omnivore density (individual birds per hectare) in *arabica* and *robusta*.

Across the three guilds, five to six of the candidate models were highly supported. For insectivores, the most important variables were pesticide use, distance to protected area, and tree cover (relative importance scores: 1, 0.71, 0.7) (Fig. 5B), while the most important variables for omnivore and frugivore abundance were distance to protected area, pesticide use, and type of coffee agroforest (Fig. 5A–C, scoring 1, 1, 0.6 for both guilds). Although the impact of environmental covariates on foraging guild density was generally unclear, omnivore densities significantly increased further from protected areas (Fig. 5). Moreover, while pesticide use was potentially detrimental for frugivore and insectivore abundance, it is possible that the density of omnivores would rise.

## Discussion

We found lower levels of forest-dependent, endemic, and foraging guild species richness in *robusta* than *arabica*. Forest-dependent, frugivorous, insectivorous, and endemic birds are sentinels of habitat quality, and are often the first taxa to disappear from modified habitats^30–32^. Yet on the whole, compared to other major cash crops in the Western Ghats such as areca (*Areca catechu*) and rubber (*Hevea brasiliensis*), Karanth *et al*.^5^ noted that coffee—aggregated across both *arabica* and *robusta*—supported higher overall avian richness, endemic richness, and greater densities for the majority of the foraging guilds as well as three out of four vertical structure guilds (low canopy, mid-, and high-canopy guilds).

*Robusta* agroforests typically supported the same or slightly higher densities of habitat specialists and foraging guilds as *arabica*, though many of these differences were not significant. This pattern was likely due to the most common forest-dependents and endemics; the observation rates for the five most common forest and endemic bird species were similar across the *arabica* and *robusta* agroforests. Existing farming practices may contribute to this outcome; only 19% of *robusta* farmers used pesticides compared to 75% of the *arabica* farmers. Reduced pesticide use in *robusta* farms could lead to increased food resources for insectivore populations. Although pesticide use did not have a clear directional impact on habitat specialist and functional guild responses, it was consistently one of the most important variables.

Our result that flock densities were slightly, but significantly, higher in *robusta* while overall habitat specialist and foraging guild richness was lower echoes recent findings that flocking behaviors may permit for habitat specialists to use more modified or disturbed habitats. Goodale *et al*.^28^ observed significant avifaunal community turnover between forest and agricultural habitat in Southern India and Sri Lanka. However, human-altered landscapes supported similar flock densities as native forest, and these mixed-species flocks appeared to recruit more forest-interior species to these more open habitats than would be otherwise expected.

Distance to protected area did not exhibit a large effect for forest and endemic bird diversity. Moreover, there was evidence that frugivore and omnivore abundance may still be high far from protected areas, suggesting that coffee agroforests can produce substantial economic and biodiversity benefits as buffers^3,5,33,34^. The pattern we observed is likely not an artifact of the range of distances to protected areas. Across the agroforests, the range of distances to protected areas extended from 0–35.9 km in *arabica* and 0.4–34.6 km in *robusta*; the maximum distances were four times farther than a comparable study in the Western Ghats^26^.

The importance of coffee agroforests as supplementary habitat may be heightened in regions with small protected areas embedded in human-use landscapes^5,19,20,23^. In other parts of tropical Asia where farming practices are more dissimilar to native forest, distance to protected area is often a significant predictor with a large impact on bird diversity^35,36^.

*Arabica* yielded higher profits per-hectare than *robusta*. Yet the planted area statistics indicated that *robusta* production has increased over the past decade. Indian *robusta* is distinguished by its high cup quality and resistance to disease, rendering it an attractive crop to farmers^37^. Additionally, certain *robusta* varieties are approaching price equalization with *arabica*^12,14–16,37^. As such, it is encouraging that *robusta* agroforests are capable of supporting abundant avian populations, both in terms of habitat specialists and foraging guilds. Nevertheless, these communities are less speciose than *arabica* assemblages.

Although our survey data did not include direct measurements of yield, there was no indication that reported pesticide use—an important determinant of production intensity—significantly affected endemic or forest-dependent avian diversity. Previous work in Southeast Asia focusing on cacao noted that yield did not necessarily correlate negatively with reduced conservation value^34^.

Managing shade tree species composition and landscape forest cover appear to be major levers for improving the biodiversity conservation value of coffee agroforests. In fact, the surveyed *robusta* agroforests possessed canopy and forest cover three times higher than shade-grown coffee farms in Indonesia and instead scored similarly to forest plots in Bukit Barisan Selatan reserve^10^. In general, the high prevalence of shade-grown coffee differentiates Indian *arabica* and *robusta* production globally, driven by historical concerns about coffee rust (*Hemileia vastatrix*)^6,38^. Ensuring the persistence of extensive forest cover at landscape scales and dense canopies of native trees would present two practical guidelines for certifying both *arabica* and *robusta*^6,10,26,33^. In highly populated landscapes such as the Western Ghats in India, and other rural tropical regions, it is critically important that calls to conserve wildlife within human-altered landscapes offer meaningful pathways to improve local people’s livelihoods that respect their aspirations^3,15,33,39^.

Certification efforts in Southeast and South Asia have largely relied on price signals which have had an equivocal impact on biodiversity conservation^6,11,19,25,38–40^. Across tropical Asia, there are repeated instances of rising coffee prices leading to clearance and conversion of protected areas or opening up of the canopy as select trees are cut down in bad years by farmers^11,41^. Wide variation across certification standards could actually incentivize the removal of shade trees that are critical for retaining habitat specialist vertebrates^23,24,42^. In fact, we observed that higher tree species richness tended to decrease the diversity of endemic birds. This is likely driven by smallholders planting exotic trees as an additional source of income; a high diversity of exotic tree species at the expense of native shade trees can be disruptive for sensitive avifauna^26,27^.

Unfortunately, recent efforts by the Rainforest Alliance to certify coffee production in the Western Ghats did not increase the conservation value of these lands; certified farmers retained on average 100 fewer native trees per hectare than non-certified producers^15^. Frequent audits and a requirement for bookkeeping may engender future hostility toward conservation interventions in this landscape, as producers expressed disappointment in measureable outcomes and certification’s limited environmental management requirements. Our research emphasizes the importance of practical recommendations for both birds and farmers.

Despite the shortcomings highlighted by Bose *et al*.^15^, due to the small median landholdings in this landscape, successful certification efforts in the Western Ghats would provide a unique and meaningful opportunity to identify management factors that are a triple win for poverty alleviation, human well-being, and conservation^38–41^. Certification often poses insurmountable financial demands for the smallest and most cash-poor farm holdings to demonstrate adherence to ecological or livelihood targets^6,11,38^.

Carbon credits as well as more rigorously audited and locally tailored management schemes could help ensure that certified coffee would be both livelihood and wildlife friendly^39–45^. It is evident that wildlife certification schemes should use scientific assessments of wildlife and be developed locally to truly enhance the value of existing coffee production systems and promote regional biodiversity. Continued work in tropical production landscapes should seek to quantify the relationship between yield, crop type, planting practice (canopy cover, tree density, retention of native trees) and a broader suite of habitat specialist taxa.

## Methods

### Social survey data

We surveyed 344 coffee agroforest owners across the three highest growing districts in Karnataka: Chikmagalur, Hassan and Kodagu^5^. More than 75% of farms in the region are <10 hectares in size. The farms selected comprised 113 *arabica* growers, 135 *robusta* growers, and 96 growing both varieties. The surveys were carried out by six trained research assistants between June 2013-July 2014.

The survey covered household demographics and socio-economics such as family size, education, income, farm size, and characteristics. Farmers were questioned about their coffee growing history in 2003, a decade before the study occurred. We also obtained details about yield and farm management, such as tree species grown, crop varieties planted, shade management and chemical inputs as well as access to institutions. Coffee production areas ranged from 2 to 250 hectares, and the range of *arabica* and *robusta* planted area was [0, 242.8 hectares] and [0, 121.4 hectares], respectively.

### Ecological data

61 coffee agroforests were surveyed for avian diversity with 30 in *arabica* and 31 in *robusta*^5^. In each farm, all point count stations were placed in only one of the crop types if both crops were grown on the farm. A minimum distance of 1 km was maintained between each sampled agroforest. Sampling occurred during the dry season (January to May 2013) and the surveys were conducted between 6:30–9:30 a.m., and 4:00–6:30 p.m, maximizing detection and visibility for passerines and near-passerines.

We evaluated bird occurrence using point counts. The number of points per farm was proportional to the size of the agroforest, ranging from 2 to 9. Each point was spaced 200 m apart for quasi-independence. At each point, two trained observers recorded all birds that were heard or sighted for 7 min after an initial wait period of 2 min to minimize the effect of disturbance. The sighting distance to each bird or clusters of birds was measured. Each point was revisited six times over three days to achieve adequate numbers of detections^46^. We sampled a total of 274 points in coffee. We excluded migrant species from our analysis to avoid biasing overall richness and density estimates. We grouped avian species into the following categories based on published sources: forest-dependent^47–49^, IUCN Red-Listed^49,50^, endemic^24,50^, and three foraging guilds—frugivorous, insectivorous, and omnivorous^51^ (Appendix II).

We examined differences between *arabica* and *robusta* management practices such as shade tree retention and species composition, coffee tree spacing, and coppicing as well as variable levels of nutrient, water and pesticide inputs (Robbins *et al*., in review). We also measured several covariates potentially associated with species occurrence including elevation, slope, weather, canopy structure, canopy density, presence of leaf litter, presence of water bodies and pesticide use^5^. Slope was measured using a compass. Canopy density was measured in all four cardinal directions at each point using a canopy densiometer. The point-centered quarter method was used to estimate tree densities at each point^52,53^.

### Analyses

#### Socioeconomic data

We calculated the mean, median, and range for planted area under each coffee species as well as the inputs used for each crop type and tree cover statistics. We identified the rate of change in acreage and proportion of planted land allocated to each coffee species. We evaluated differences in means using Wilcoxon Rank Sum tests. We applied Bonferroni familywise-error adjustment.

The avifaunal point count stations were matched to farms using a unique identifier, in order to associate the point count locations with environmental and farming practices covariates.

#### Ecological data

The asymptotic richness and community composition of forest-dependent, endemic, and threatened birds as well as the three foraging guilds was calculated using the package *vegan* (v 2.4.1) in R (v 3.3.1) at the level of individual agroforests and across crop types^54,55^. We determined the abundance of individual birds and flocks using the package *Distance* (v 0.9.6) in R^56^. Crop type (*arabica* versus *robusta*) was defined as the region, and the total area for each region was summed across all point count stations within each crop type, assuming that each point count had a radius of 100 m. The individual samples were the point count stations; as such, effort was the number of visits to each station. Truncation was performed at 100 m.

We used asymptotic richness estimates as response variables for the habitat specialists, while the estimated densities of foraging guilds were used as the modelled response. We constructed generalized linear models with a Gaussian error distribution to ascertain the relationship between avian habitat specialist diversity or foraging guild abundance and several habitat and farming practice covariates. All predictor variables were normalized and showed no evidence of multicollinearity. For the habitat specialist richness regressions, the survey effort at each farm (the number of point count stations per farm in this case) was supplied as an offset. Model suitability was visually assessed using diagnostic residual, Q-Q, and leverage plots.

We performed multimodel inference to evaluate empirical support for several hypotheses related to crop type, non-coffee tree cover and species richness, non-coffee tree density, canopy density, canopy structure, distance to the nearest protected area, and pesticide usage. A total of eight candidate models were compared using the package *MuMIn* in R (v 1.15.6)^57^. We performed full model averaging with a shrinkage estimator across the most parsimonious candidates (ΔAICc ≤4)^58^. We evaluated whether or not there was evidence of residual spatial autocorrelation using Moran’s I with the R packages *ape* (v 4.1) and *geosphere* (v 1.5-5)^59,60^.

### Data archiving statement

The ecological data have been formatted for replication analyses in R, and saved as an.Rdata file with an accompanying R script for running the relevant analyses, as well as a description of each object. These objects can be accessed in the article’s Supplementary Information and are mirrored at https://github.com/charlottehchang/WCS-India-Coffee. The appendix details how to access the replication data and perform analyses. Due to legal and ethical constraints given the sensitivity of surveying farmers in India, we are unable to provide the socio-economic household data.

## Electronic supplementary material

Supplementary Information
