# Author Correction: Birds and beans: Comparing avian richness and endemism in *arabica* and *robusta* agroforests in India’s Western Ghats

**DOI:** 10.1038/s41598-018-23597-8

**Published:** 2018-04-11

**Authors:** Charlotte H. Chang, Krithi K. Karanth, Paul Robbins

**Affiliations:** 1 Department of Ecology and Evolutionary Biology, Princeton University, Princeton, NJ 08544 USA; 2 Wildlife Conservation Society, 2300 Bronx Blvd, New York, USA; 3 Centre for Wildlife Studies, 551, 7th Main Road Rajiv Gandhi Nagar, 2nd Phase, Kodigehalli, Bengaluru, 560097 India; 4 Duke University, Durham, North Carolina USA; 5 Nelson Institute, University of Wisconsin-Madison, Madison, Wisconsin USA

Correction to: *Scientific Reports* 10.1038/s41598-018-21401-1, published online 16 February 2018

In this Article, the authors neglected to include some potential conflicts of interest. The Competing Interests section should read:

**“**This paper uses bird data from 61 coffee farms collected in 2013–2014 and supported by the NSF project to Dr. Robbins, Karanth and Chhatre. In 2017 Dr. Karanth co-founded Wild Kaapi, a wildlife friendly coffee company sources coffee and independently audits for birds, butterflies, trees, mammals and amphibians. Dr’s Paul Robbins and Charlotte Chang are not associated with Wild Kaapi.”

Additionally, this Article contains typographical errors in the Acknowledgements section.

“Funding for this project was provided by US NSF Grant Number 1265223 (PR, KKK, AC Chhhatre), Oracle (KKK), the US NSF Graduate Research Fellowship (CHC) and Graduate Research Opportunities Worldwide with USAID program (CHC).”

should read:

“Funding for this project was provided by US NSF Grant Number 1265223 (PR, KKK, A Chhatre), Oracle (KKK), the US NSF Graduate Research Fellowship (CHC) and Graduate Research Opportunities Worldwide with USAID program (CHC).”

“We thank Vishnupriya Sankararaman and Shashank Dalvi for leading the field efforts, and volunteers who data.”

should read:

“We thank Vishnupriya Sankararaman and Shashank Dalvi for leading the field efforts, and volunteers who collected data.”

