# Supplementary material for: Birds and beans: Comparing avian richness and endemism in arabica and robusta agroforests in India’s Western Ghats
Source: Sci Rep. 2018 Feb 16;8:3143. doi: 10.1038/s41598-018-21401-1 (PMC5816607; doi:10.1038/s41598-018-21401-1)
Supplement: Supplementary file 1 — Supplementary Information [file 41598_2018_21401_MOESM1_ESM.pdf]

Appendix to Birds and beans: Comparing avian richness and endemism in *arabica* and *robusta* agroforests in India's Western Ghats

Charlotte H. Chang[1], Krithi K. Karanth[*,2,3,4], Paul Robbins[5]

[1]: Department of Ecology and Evolutionary Biology, Princeton University [2]: Wildlife Conservation Society, 2300 Bronx Blvd, New York, USA [3]: Centre for Wildlife Studies, No. 1669, 31st Cross, 16th Main, Banashankari 2nd Stage, Bangalore, India [4]: Duke University, Durham, North Carolina, USA [5]: Nelson Institute, University of Wisconsin-Madison

[*]Corresponding author: Krithi K. Karanth[2,3,4], krithi.karanth@gmail.com

Running headline: *Avian endemism and richness in* arabica *and* robusta *agroforests*

Table A1: The five most common species of each category observed in arabica and robusta plantations.

Crop	Forest-dependent	Endemic	Frugivore	Insectivore	Omnivore
Arabica	Cinnyris lotenius (100)	Ocyceros griseus (77.2)	Cinnyris lotenius (100)	Vanellus indicus (99.4)	Amaurornis phoenicurus (100)
	Cuculus micropterus (85.6)	Psilopogon malabaricus (41.1)	Ocyceros griseus (77.2)	Copsychus saularis (86.1)	Parus major (67.8)
	Ocyceros griseus (77.2)	Turdoides subrufa (27.2)	Dicaeum agile (59.4)	Cuculus micropterus (85.6)	Acridotheres tristis (60)
	Sitta frontalis (75.6)	Pycnonotus gularis (20)	Dicaeum erythrorhynchos (56.1)	Sitta frontalis (75.6)	Coracias benghalensis (53.3)
	Hemicircus canente (68.9)	Galloperdix spadicea (10.6)	Columba elphinstonii (46.7)	Hemicircus canente (68.9)	Centropus sinensis (37.8)
Robusta	Cinnyris lotenius (96.7)	Ocyceros griseus (72.8)	Cinnyris lotenius (96.7)	Vanellus indicus (101.7)	Amaurornis phoenicurus (103.3)
	Cuculus micropterus (78.9)	Psilopogon malabaricus (40.6)	Ocyceros griseus (72.8)	Cuculus micropterus (78.9)	Parus major (77.8)
	Sitta frontalis (78.3)	Pycnonotus gularis (22.2)	Dicaeum erythrorhynchos (69.4)	Sitta frontalis (78.3)	Acridotheres tristis (51.1)
	Hemicircus canente (77.8)	Turdoides subrufa (19.4)	Psittacula cyanocephala (43.9)	Hemicircus canente (77.8)	Coracias benghalensis (50)
	Chrysocolaptes guttacristatus (73.3)	Galloperdix spadicea (7.2)	Columba elphinstonii (41.1)	Chrysocolaptes guttacristatus (73.3)	Centropus sinensis (34.4)

*: User% refers to the percentage of surveyed growers for each coffee strain that use this particular input; pay % is the proportion of respondents who paid for these inputs. Mean (standard error) refers to the average amount of money (2013 $USD) per hectare paid per input, and range shows the minimum and maximum per hectare costs associated with each input.

Table A2: Forest-dependent richness model comparison

Variables (β (SE))	Models							
	(1)	(2)	(3)	(4)	(5)	(6)	(7)	(8)
Crop: Robusta	0.33 (2.77)	-0.58 (2.58)				0.76 (2.28)	0.27 (2.24)	0.35 (2.26)
Tree richness	0.53 (1.10)	0.40 (1.08)						0.55 (1.00)
Tree density	-0.03 (1.22)	-0.36 (1.15)	-0.49 (1.05)	-0.39 (1.08)				
Canopy density	-1.67 (1.37)	-1.22 (1.27)	-1.03 (1.14)	-1.06 (1.15)				
Canopy structure	1.10 (1.21)							
Tree cover	1.43 (1.08)	1.33 (1.04)	1.37 (1.02)	1.30 (1.04)	1.02 (1.00)	1.09 (1.02)		
Distance to PA	-0.51 (1.11)			-0.44 (1.09)	-0.57 (1.05)	-0.58 (1.06)	-0.77 (1.05)	-0.73 (1.06)
Pesticide use	0.68 (1.25)	0.38 (1.17)	0.55 (1.05)	0.69 (1.11)	0.30 (1.04)	0.50 (1.20)	0.32 (1.19)	0.23 (1.21)
Constant	30.10*** (1.72)	30.56*** (1.64)	30.27*** (0.96)	30.27*** (0.97)	30.27*** (0.96)	29.88*** (1.51)	30.13*** (1.50)	30.09*** (1.51)
N	61	61	61	61	61	61	61	61
Log Likelihood	-207.29	-207.86	-207.97	-207.88	-208.64	-208.58	-209.19	-209.03
AIC	432.57	429.72	425.95	427.77	425.28	427.16	426.38	428.05

$^{*}p < .1$; $^{**}p < .05$; $^{***}p < .01$

Table A3: Endemic richness model comparison

	Model							
Variables (β (SE))	(1)	(2)	(3)	(4)	(5)	(6)	(7)	(8)
Crop: Robusta	0.8 (0.8)	1.0 (0.8)				1.0 (0.8)	0.9 (0.8)	0.7 (0.7)
Tree richness	-1.5*** (0.3)	-1.5*** (0.3)						-1.5*** (0.3)
Tree density	-0.03 (0.4)	0.1 (0.4)	0.6 (0.4)	0.6 (0.4)				
Canopy density	0.3 (0.4)	0.2 (0.4)	-0.3 (0.4)	-0.3 (0.4)				
Canopy structure	-0.3 (0.4)							
Tree cover	0.1 (0.3)	0.1 (0.3)	-0.01 (0.4)	0.01 (0.4)	-0.05 (0.4)	0.04 (0.4)		
Distance to PA	0.2 (0.3)			0.2 (0.4)	0.3 (0.4)	0.3 (0.4)	0.3 (0.4)	0.2 (0.3)
Pesticide use	-0.2 (0.4)	-0.1 (0.4)	-0.5 (0.4)	-0.5 (0.4)	-0.6 (0.4)	-0.4 (0.4)	-0.4 (0.4)	-0.2 (0.4)
Constant	-0.2 (0.5)	-0.4 (0.5)	0.2 (0.3)	0.2 (0.4)	0.2 (0.4)	-0.3 (0.5)	-0.3 (0.5)	-0.2 (0.5)
N	61	61	61	61	61	61	61	61
Log Likelihood	-135.2	-135.7	-146.1	-146.0	-147.1	-146.4	-146.4	-135.8
AIC	288.4	285.5	302.2	304.0	302.3	302.8	300.8	281.6

$^{*}p < .1$; $^{**}p < .05$; $^{***}p < .01$

Table A4: Frugivore density model comparison

	Model							
Variables (β (SE))	(1)	(2)	(3)	(4)	(5)	(6)	(7)	(8)
Crop: Robusta	1.1 (0.9)	1.1 (0.9)				0.7 (0.8)	0.6 (0.7)	0.7 (0.7)
Tree richness	0.3 (0.4)	0.3 (0.4)						0.4 (0.3)
Tree density	-0.2 (0.4)	-0.1 (0.4)	-0.3 (0.4)	-0.5 (0.4)				
Canopy density	0.3 (0.5)	0.3 (0.4)	0.2 (0.4)	0.2 (0.4)				
Canopy structure	0.3 (0.4)							
Tree cover	0.2 (0.4)	0.1 (0.4)	0.04 (0.4)	0.2 (0.3)	0.2 (0.3)	0.3 (0.3)		
Distance to PA	0.7^{*} (0.4)			0.8^{**} (0.4)	0.7^{*} (0.3)	0.6^{*} (0.3)	0.6^{*} (0.3)	0.6^{*} (0.3)
Pesticide use	-0.1 (1.6)	0.7 (1.6)	0.1 (1.4)	-0.8 (1.5)	-0.6 (1.4)	0.2 (1.6)	-0.005 (1.6)	-0.3 (1.6)
Constant	4.0^{***} (0.7)	3.9^{***} (0.7)	4.6^{***} (0.4)	4.7^{***} (0.4)	4.7^{***} (0.4)	4.2^{***} (0.6)	4.3^{***} (0.6)	4.3^{***} (0.6)
N	61	61	61	61	61	61	61	61
Log Likelihood	-139.3	-141.7	-142.9	-140.5	-141.4	-140.9	-141.3	-140.4
AIC	296.6	297.5	295.8	293.0	290.8	291.8	290.5	290.8

$^{*}p < .1$; $^{**}p < .05$; $^{***}p < .01$

Table A5: Insectivore density model comparison

	Model							
Variables (β (SE))	(1)	(2)	(3)	(4)	(5)	(6)	(7)	(8)
Crop: Robusta	-0.1 (0.5)	0.1 (0.5)				0.4 (0.4)	0.3 (0.4)	0.3 (0.4)
Tree richness	-0.1 (0.2)	-0.03 (0.2)						0.04 (0.2)
Tree density	-0.3 (0.2)	-0.2 (0.2)	-0.2 (0.2)	-0.2 (0.2)				
Canopy density	-0.2 (0.2)	-0.2 (0.2)	-0.3 (0.2)	-0.2 (0.2)				
Canopy structure	-0.2 (0.2)							
Tree cover	0.1 (0.2)	0.1 (0.2)	0.1 (0.2)	0.2 (0.2)	0.1 (0.2)	0.1 (0.2)		
Distance to PA	0.2 (0.2)			0.2 (0.2)	0.1 (0.2)	0.1 (0.2)	0.1 (0.2)	0.1 (0.2)
Pesticide use	-0.4 (0.9)	-0.1 (0.8)	-0.2 (0.8)	-0.4 (0.8)	-0.8 (0.8)	-0.4 (0.9)	-0.5 (0.9)	-0.5 (0.9)
Constant	2.9^{***} (0.4)	2.8^{***} (0.4)	2.8^{***} (0.2)	2.9^{***} (0.2)	2.9^{***} (0.2)	2.7^{***} (0.4)	2.7^{***} (0.3)	2.7^{***} (0.3)
N	61	61	61	61	61	61	61	61
Log Likelihood	-102.1	-102.9	-103.0	-102.5	-104.6	-104.2	-104.5	-104.4
AIC	222.2	219.9	216.0	217.0	217.2	218.3	216.9	218.9

$^{*}p < .1$; $^{**}p < .05$; $^{***}p < .01$

Table A6: Omnivore density model comparison

Variables (β (SE))	Model (1)	(2)	(3)	(4)	(5)	(6)	(7)	(8)
Crop: Robusta	0.2 (0.7)	0.5 (0.7)				0.8 (0.6)	0.9 (0.6)	0.8 (0.6)
Tree richness	-0.3 (0.3)	-0.3 (0.3)						-0.1 (0.3)
Tree density	-0.6^{*} (0.3)	-0.4 (0.3)	-0.3 (0.3)	-0.5 (0.3)				
Canopy density	0.02 (0.4)	-0.1 (0.4)	-0.2 (0.3)	-0.2 (0.3)				
Canopy structure	-0.2 (0.3)							
Tree cover	-0.2 (0.3)	-0.3 (0.3)	-0.3 (0.3)	-0.2 (0.3)	-0.3 (0.3)	-0.2 (0.3)		
Distance to PA	0.7^{**} (0.3)			0.7^{**} (0.3)	0.6^{**} (0.3)	0.6^{**} (0.3)	0.6^{**} (0.3)	0.6^{**} (0.3)
Pesticide use	0.9 (1.3)	2.0 (1.3)	1.5 (1.2)	0.6 (1.2)	0.2 (1.1)	1.0 (1.3)	1.2 (1.3)	1.3 (1.3)
Constant	3.9^{***} (0.6)	3.6^{***} (0.6)	3.9^{***} (0.3)	4.0^{***} (0.3)	4.1^{***} (0.3)	3.6^{***} (0.5)	3.5^{***} (0.5)	3.5^{***} (0.5)
N	61	61	61	61	61	61	61	61
Log Likelihood	-126.4	-129.9	-130.7	-127.5	-129.8	-129.0	-129.3	-129.2
AIC	270.9	273.7	271.4	267.0	267.6	268.0	266.6	268.3

$^{*}p < .1$; $^{**}p < .05$; $^{***}p < .01$

Appendix II: List of habitat specialization traits for all surveyed species

Table 1: Threat and endemism status for observed birds.

Original	Latin	Migratory	Threat	Endemism
Alexandrine Parakeet	Psittacula eupatria	0	NT	0
Grey-headed Bulbul	Pycnonotus priocephalus	0	NT	1
Flame-throated Bulbul	Pycnonotus gularis	0	LC	1
Malabar Woodshrike	Tephrodornis sylvicola	0	LC	1
Nilgiri Flowerpecker	Dicaeum concolor	0	LC	1
Red Spurfowl	Galloperdix spadicea	0	LC	1
Grey Junglefowl	Gallus sonneratii	0	LC	1
Malabar Barbet	Psilopogon malabaricus	0	LC	1
Malabar Grey Hornbill	Ocyceros griseus	0	LC	1
Malabar Parakeet	Psittacula columboides	0	LC	1
Malabar Whistling Thrush	Myophonus horsfieldii	0	LC	1
Rufous Babbler	Turdoides subrufa	0	LC	1
Small Sunbird	Leptocoma minima	0	LC	1
White-bellied Blue-flycatcher	Cyornis pallipes	0	LC	1
White-cheeked Barbet	Psilopogon viridis	0	LC	1
Malabar Trogon	Harpactes fasciatus	0	LC	0
Nilgiri Wood-pigeon	Columba elphinstonii	0	VU	0
Collared Scops Owl	Otus lettia	0	LC	0
Common Flameback	Dinopium javanense	0	LC	0
Common Hawk Cuckoo	Hierococcyx varius	0	LC	0
Common Iora	Aegithina tiphia	0	LC	0
Common Myna	Acridotheres tristis	0	LC	0

Common Tailorbird	Orthotomus sutorius	0	LC	0
Common Woodshrike	Tephrodornis pondicerianus	0	LC	0
Coppersmith Barbet	Psilopogon haemacephalus	0	LC	0
Crested Goshawk	Accipiter trivirgatus	0	LC	0
Crested Serpent Eagle	Spilornis cheela	0	LC	0
Dark-fronted Babbler	Rhopocichla atriceps	0	LC	0
Drongo Cuckoo	Surniculus dicruroides	0	LC	0
Eastern Cattle Egret	Bubulcus ibis	0	LC	0
Emerald Dove	Chalcophaps indica	0	LC	0
Golden Oriole	Oriolus kundoo	0	LC	0
Ashy Wood Swallow	Artamus fuscus	0	LC	0
Asian Fairy Bluebird	Irena puella	0	LC	0
Asian Koel	Eudynamys scolopaceus	0	LC	0
Asian Paradise Flycatcher	Terpsiphone paradisi	0	LC	0
Banded Bay Cuckoo	Cacomantis sonneratii	0	LC	0
Bar-winged Flycatchershrike	Hemipus picatus	0	LC	0
Black Drongo	Dicrurus macrocercus	0	LC	0
Black-headed Cuckooshrike	Coracina melanoptera	0	LC	0
Black-hooded Oriole	Oriolus xanthornus	0	LC	0
Black-lored Tit	Parus xanthogenys	0	LC	0
Black-naped Monarch	Hypothymis azurea	0	LC	0
Black-rumped Flameback	Dinopium benghalense	0	LC	0
Black-shouldered Kite	Elanus caeruleus	0	LC	0
Black-throated Munia	Lonchura kelaarti	0	LC	0

Blue-bearded Bee-eater	Nyctyornis athertoni	0	LC	0
Brahminy Myna	Sturnia pagodarum	0	LC	0
Bronzed Drongo	Dicrurus aeneus	0	LC	0
Brown Fish Owl	Ketupa zeylonensis	0	LC	0
Brown Hawk Owl	Ninox scutulata	0	LC	0
Brown-capped Pygmy Woodpecker	Yungipicus nanus	0	LC	0
Brown-cheeked Fulvetta	Alcippe poioicephala	0	LC	0
Chestnut-headed Bee-eater	Merops leschenaulti	0	LC	0
Chestnut-shouldered Petronia	Gymnoris xanthocollis	0	LC	0
Golden-fronted Leafbird	Chloropsis aurifrons	0	LC	0
Great Tit	Parus major	0	LC	0
Greater Coucal	Centropus sinensis	0	LC	0
Greater Flameback	Chrysocolaptes guttacristatus	0	LC	0
Greater Racket-tailed Drongo	Dicrurus paradiseus	0	LC	0
Grey-bellied Cuckoo	Cacomantis passerinus	0	LC	0
Grey-fronted Green Pigeon	Treron affinis	0	LC	0
Heart-spotted Woodpecker	Hemicircus canente	0	LC	0
Hill Myna	Gracula indica	0	LC	0
Indian Cormorant	Phalacrocorax fuscicollis	0	LC	0
Indian Cuckoo	Cuculus micropterus	0	LC	0
Indian Jungle Crow	Corvus macrorhynchos	0	LC	0
Indian Nuthatch	Sitta castanea	0	LC	0
Indian Peafowl	Pavo cristatus	0	LC	0
Indian Pond Heron	Ardeola grayii	0	LC	0

Indian Roller	Coracias benghalensis	0	LC	0
Indian Scimitar Babbler	Pomatorhinus horsfieldii	0	LC	0
Jerdons Leafbird	Chloropsis jerdoni	0	LC	0
Jungle Babbler	Turdoides striata	0	LC	0
Jungle Myna	Acridotheres fuscus	0	LC	0
Jungle Owlet	Glaucidium radiatum	0	LC	0
Large Cuckooshrike	Coracina macei	0	LC	0
Lesser Yellownape	Picus chlorolophus	0	LC	0
Little Egret	Egretta garzetta	0	LC	0
Little Spiderhunter	Arachnothera longirostra	0	LC	0
Long-tailed Shrike	Lanius schach	0	LC	0
Lotens Sunbird	Cinnyris lotenius	0	LC	0
Mottled Wood Owl	Strix ocellata	0	LC	0
Orange minivet	Pericrocotus flammeus	0	LC	0
Orange-headed Thrush	Zoothera citrina	0	LC	0
Oriental Honey Buzzard	Pernis ptilorhynchus	0	LC	0
Oriental Magpie Robin	Copsychus saularis	0	LC	0
Oriental White Eye	Zosterops palpebrosus	0	LC	0
Pale-billed Flowerpecker	Dicaeum erythrorhynchos	0	LC	0
Plum-headed Parakeet	Psittacula cyanocephala	0	LC	0
Puff-throated Babbler	Pellorneum ruficeps	0	LC	0
Purple Sunbird	Cinnyris asiaticus	0	LC	0
Purple-rumped Sunbird	Leptocoma zeylonica	0	LC	0
Red-vented Bulbul	Pycnonotus cafer	0	LC	0
Red-wattled Lapwing	Vanellus indicus	0	LC	0

Red-whiskered Bulbul	Pycnonotus jocosus	0	LC	0
Rock Pigeon	Columba livia	0	LC	0
Rose-ringed Parakeet	Psittacula krameri	0	LC	0
Rufous Treepie	Dendrocitta vagabunda	0	LC	0
Rufous Woodpecker	Micropternus brachyurus	0	LC	0
Shikra	Accipiter badius	0	LC	0
Small Green Bee-eater	Merops orientalis	0	LC	0
Small Minivet	Pericrocotus cinnamomeus	0	LC	0
Spangled Drongo	Dicrurus hottentottus	0	LC	0
Speckled Piculet	Picumnus innominatus	0	LC	0
Spotted Dove	Spilopelia suratensis	0	LC	0
Square-tailed Black Bulbul	Hypsipetes ganeesa	0	LC	0
Thick-billed Flowerpecker	Dicaeum agile	0	LC	0
Tickells Blue Flycatcher	Cyornis tickelliae	0	LC	0
Velvet-fronted Nuthatch	Sitta frontalis	0	LC	0
Vernal Hanging Parrot	Loriculus vernalis	0	LC	0
White-bellied Woodpecker	Dryocopus javensis	0	LC	0
White-breasted Waterhen	Amaurornis phoenicurus	0	LC	0
White-rumped Munia	Lonchura striata	0	LC	0
White-rumped Shama	Copsychus malabaricus	0	LC	0
White-throated Kingfisher	Halcyon smyrnensis	0	LC	0
Yellow-browed Bulbul	Acritillas indica	0	LC	0
Yellow-crowned Woodpecker	Leiopicus mahrattensis	0	LC	0
Yellow-footed Green Pigeon	Treron phoenicopterus	0	LC	0

Table 2: Forest-dependency of the observed avifauna.

Scientific Name	Category	Source
Dicrurus leucophaeus	5	Anand
Monticola cinclorhynchus	5	Anand
Muscicapa dauurica	5	Anand
Eumyias thalassina	5	Anand
Luscinia brunnea	5	Anand
Acrocephalus dumetorum	5	Anand
Phylloscopus trochiloides	5	Anand
Phylloscopus magnirostris	5	Anand
Phylloscopus occipitalis	5	Anand
Dendronanthus indicus	5	Anand
Motacilla cinerea	5	Anand
Carpodacus erythrinus	5	Anand
Psilopogon viridis	1	Anand
Psilopogon rubricapilla	1	Anand
Ocyceros griseus	1	Anand
Harpactes fasciatus	1	Anand
Psittacula columboides	1	Anand
Dendrocitta leucogastra	1	Anand
Myophonus horsfieldii	1	Anand
Cyornis pallipes	1	Anand
Acritillas indica	1	Anand
Pomatorhinus horsfieldii	1	Anand
Rhopocichla atriceps	1	Anand

Turdoides subrufa	1	Anand
Leptocoma minima	1	Anand
Cinnyris lotenius	1	Anand
Lonchura kelaarti	1	Anand
Picumnus innominatus	2	Anand
Hemicircus canente	2	Anand
Dinopium javanense	2	Anand
Ducula badia	2	Anand
Treron affinis	2	Anand
Accipiter trivirgatus	2	Anand
Accipiter virgatus	2	Anand
Irena puella	2	Anand
Tephrodornis gularis	2	Anand
Pycnonotus melanicterus	2	Anand
Hypsipetes ganeesa	2	Anand
Dicaeum concolor	2	Anand
Arachnothera longirostra	2	Anand
Galloperdix spadicea	3	Anand
Gallus sonneratii	3	Anand
Celeus brachyurus	3	Anand
Dryocopus javensis	3	Anand
Yungipicus nanus	3	Anand
Leiopicus mahrattensis	3	Anand
Picus chlorolophus	3	Anand
Chrysocolaptes guttacristatus	3	Anand

Merops leschenaulti	3	Anand
Cacomantis sonneratii	3	Anand
Centropus sinensis	3	Anand
Loriculus vernalis	3	Anand
Psittacula cyanocephala	3	Anand
Bubo bubo	3	Anand
Chalcophaps indica	3	Anand
Spilornis cheela	3	Anand
Chloropsis jerdoni	3	Anand
Chloropsis aurifrons	3	Anand
Dendrocitta vagabunda	3	Anand
Oriolus kundoo	3	Anand
Pericrocotus cinnamomeus	3	Anand
Pericrocotus flammeus	3	Anand
Hemipus picatus	3	Anand
Dicrurus aeneus	3	Anand
Dicrurus hottentottus	3	Anand
Dicrurus paradiseus	3	Anand
Hypothymis azurea	3	Anand
Terpsiphone paradisi	3	Anand
Zoothera citrina	3	Anand
Turdus merula	3	Anand
Cyornis tickelliae	3	Anand
Culicicapa ceylonensis	3	Anand
Copsychus malabaricus	3	Anand

Sturnus malabaricus	3	Anand
Gracula indica	3	Anand
Sitta frontalis	3	Anand
Sitta castanea	3	Anand
Parus xanthogenys	3	Anand
Pellorneum ruficeps	3	Anand
Alcippe poioicephala	3	Anand
Dicaeum agile	3	Anand
Gymnoris xanthocollis	3	Anand
Pavo cristatus	4	Anand
Dinopium benghalense	4	Anand
Upupa epops	4	Anand
Halcyon smyrnensis	4	Anand
Psittacula krameri	4	Anand
Streptopelia chinensis	4	Anand
Accipiter badius	4	Anand
Corvus macrorhynchos	4	Anand
Coracina macei	4	Anand
Aegithina tiphia	4	Anand
Copsychus saularis	4	Anand
Acridotheres fuscus	4	Anand
Pycnonotus jocosus	4	Anand
Pycnonotus cafer	4	Anand
Prinia socialis	4	Anand
Zosterops palpebrosus	4	Anand

Orthotomus sutorius	4	Anand
Turdoides striata	4	Anand
Dicaeum erythrorhynchos	4	Anand
Cinnyris asiaticus	4	Anand
Abroscopus superciliaris	4	Beukema
Accipiter gularis	5	Beukema
Accipiter trivirgatus	3	Beukema
Aegithina tiphia	4	Beukema
Aegithina viridissima	3	Beukema
Aethopyga siparaja	4	Beukema
Alcippe brunneicauda	3	Beukema
Amaurornis phoenicurus	5	Beukema
Anorrhinus galeritus	3	Beukema
Anthracoceros albirostris	4	Beukema
Anthracoceros malayanus	4	Beukema
Anthreptes malacensis	5	Beukema
Anthreptes rhodolaema	4	Beukema
Anthreptes simplex	3	Beukema
Anthreptes singalensis	4	Beukema
Aplonis panayensis	5	Beukema
Arachnothera affinis	3	Beukema
Arachnothera chrysogenys	3	Beukema
Arachnothera flavigaster	3	Beukema
Arachnothera longirostra	3	Beukema
Arachnothera robusta	3	Beukema

Argusianus argus	3	Beukema
Aviceda jerdoni	3	Beukema
Blythipicus rubiginosus	3	Beukema
Buceros rhinoceros	3	Beukema
Cacomantis merulinus	5	Beukema
Cacomantis sonneratii	4	Beukema
Calorhamphus fuliginosus	4	Beukema
Calyptomena viridis	3	Beukema
Micropternus brachyurus	4	Beukema
Centropus bengalensis	5	Beukema
Centropus sinensis	5	Beukema
Cettia vulcania	3	Beukema
Ceyx erithacus	3	Beukema
Chalcophaps indica	3	Beukema
Chloropsis cochinchinensis	4	Beukema
Chloropsis cyanopogon	4	Beukema
Chloropsis sonnerati	3	Beukema
Chrysococcyx xanthorhynchus	4	Beukema
Copsychus malabaricus	3	Beukema
Copsychus saularis	5	Beukema
Coracina striata	3	Beukema
Corvus enca	4	Beukema
Corvus macrorhynchos	5	Beukema
Criniger bres	3	Beukema
Criniger ochraceus	3	Beukema

Criniger phaeocephalus	3	Beukema
Culicicapa ceylonensis	3	Beukema
Cymbirhynchus macrorhynchos	4	Beukema
Cyornis tickelliae	4	Beukema
Cyornis turcosus	3	Beukema
Dicaeum cruentatum	4	Beukema
Dicaeum trigonostigma	4	Beukema
Dicrurus aeneus	4	Beukema
Dicrurus paradiseus	4	Beukema
Dinopium javanense	4	Beukema
Dinopium rafflesii	3	Beukema
Dryocopus javensis	3	Beukema
Ducula aenea	3	Beukema
Eupetes macrocerus	3	Beukema
Eurylaimus javanicus	3	Beukema
Eurylaimus ochromalus	4	Beukema
Eurystomus orientalis	4	Beukema
Gallus gallus	4	Beukema
Gracula religiosa	4	Beukema
Halcyon chloris	5	Beukema
Halcyon smyrnensis	5	Beukema
Harpactes diardii	3	Beukema
Harpactes duvaucelii	3	Beukema
Harpactes kasumba	3	Beukema
Harpactes orrhophaeus	3	Beukema

Hemicircus concretus	4	Beukema
Hemiprocne comata	4	Beukema
Hemipus hirundinaceus	4	Beukema
Hypogramma hypogrammicum	3	Beukema
Hypothymis azurea	3	Beukema
Hypsipetes charlottae	3	Beukema
Hypsipetes criniger	3	Beukema
Irena puella	3	Beukema
Kenopia striata	3	Beukema
Lalage nigra	5	Beukema
Lanius cristatus	3	Beukema
Lonchura leucogastra	4	Beukema
Lonchura striata	5	Beukema
Lophura erythrophthalma	3	Beukema
Loriculus galgulus	4	Beukema
Macronous gularis	4	Beukema
Macronous ptilosus	3	Beukema
Malacopteron affine	3	Beukema
Malacopteron cinereum	3	Beukema
Malacopteron magnirostre	3	Beukema
Malacopteron magnum	3	Beukema
Megalaima australis	4	Beukema
Megalaima chrysopogon	3	Beukema
Psilopogon haemacephalus	5	Beukema
Megalaima henricii	3	Beukema

Megalaima mystacophanos	3	Beukema
Megalaima rafflesii	3	Beukema
Meiglyptes tristis	4	Beukema
Meiglyptes tukki	3	Beukema
Merops viridis	5	Beukema
Microhierax fringillarius	4	Beukema
Napothera macrodactyla	3	Beukema
Nectarinia jugularis	5	Beukema
Nectarinia sperata	4	Beukema
Nyctyornis amictus	3	Beukema
Oriolus chinensis	5	Beukema
Oriolus xanthonotus	3	Beukema
Orthotomus atrogularis	5	Beukema
Orthotomus ruficeps	5	Beukema
Orthotomus sericeus	4	Beukema
Pelargopsis capensis	5	Beukema
Pellorneum capistratum	3	Beukema
Pericrocotus cinnamomeus	4	Beukema
Pericrocotus flammeus	3	Beukema
Philentoma pyrhopterum	3	Beukema
Philentoma velatum	3	Beukema
Picus puniceus	3	Beukema
Platylophus galericulatus	3	Beukema
Platysmurus leucopterus	3	Beukema
Pomatorhinus montanus	3	Beukema

Prinia atrogularis *	4	Beukema
Prinia familiaris	5	Beukema
Prinia flaviventris	5	Beukema
Prionochilus maculatus	3	Beukema
Prionochilus percussus	3	Beukema
Psittacula longicauda	4	Beukema
Psittinus cyanurus	3	Beukema
Ptilinopus jambu	3	Beukema
Pycnonotus atriceps	4	Beukema
Pycnonotus brunneus	3	Beukema
Pycnonotus cyaniventris	3	Beukema
Pycnonotus erythropthalmos	4	Beukema
Pycnonotus eutilotus	3	Beukema
Pycnonotus goiavier	5	Beukema
Pycnonotus melanicterus	4	Beukema
Pycnonotus melanoleucos	3	Beukema
Pycnonotus plumosus	5	Beukema
Pycnonotus simplex	3	Beukema
Reinwardtipicus validus	3	Beukema
Rhamphococcyx curvirostris	4	Beukema
Rhinomyias olivacea	3	Beukema
Rhinomyias umbratilis	3	Beukema
Rhinoplax vigil	3	Beukema
Rhinortha chlorophaea	4	Beukema
Rhopodytes diardi	4	Beukema

Rhopodytes sumatranus	3	Beukema
Rhyticeros corrugatus	3	Beukema
Rhyticeros undulatus	3	Beukema
Sasia abnormis	4	Beukema
Spilornis cheela	4	Beukema
Stachyris erythroptera	3	Beukema
Stachyris maculata	3	Beukema
Stachyris nigricollis	3	Beukema
Stachyris poliocephala	3	Beukema
Stachyris rufifrons	3	Beukema
Surniculus dicruroides	4	Beukema
Terpsiphone paradisi	3	Beukema
Treron olax	4	Beukema
Treron vernans	4	Beukema
Trichastoma abbotti	3	Beukema
Trichastoma bicolor	3	Beukema
Trichastoma malaccense	3	Beukema
Trichastoma pyrrogenys	3	Beukema
Trichastoma sepiarium	3	Beukema
Zanclostomus javanicus	4	Beukema
Bubulcus ibis	4	Chang
Artamus fuscus	4	Chang
Dicrurus macrocercus	4	Chang
Parus major	4	Chang
Phalacrocorax fuscicollis	4	Chang

Ardeola grayii	4	Chang
Coracias benghalensis	4	Chang
Egretta garzetta	4	Chang
Lanius schach	4	Chang
Leptocoma zeylonica	4	Chang
Vanellus indicus	4	Chang
Columba livia	4	Chang
Spilopelia suratensis	4	Chang
Eudynamys scolopaceus	3	Chang
Nyctyornis athertoni	3	Chang
Cuculus micropterus	3	Chang
Pernis ptilorhynchus	3	Chang
Merops orientalis	3	Chang
Glaucidium radiatum	2	Chang
Psittacula eupatria	3	Chang
Pycnonotus priocephalus	2	Chang
Pycnonotus gularis	3	Chang
Tephrodornis sylvicola	3	Chang
Psilopogon malabaricus	2	Chang
Columba elphinstonii	2	Chang
Otus lettia	4	Chang
Hierococcyx varius	3	Chang
Acridotheres tristis	4	Chang
Tephrodornis pondicerianus	3	Chang
Coracina melanoptera	3	Chang

Oriolus xanthornus	3	Chang
Elanus caeruleus	3	Chang
Sturnia pagodarum	3	Chang
Ketupa zeylonensis	2	Chang
Ninox scutulata	3	Chang
Cacomantis passerinus	4	Chang
Strix ocellata	4	Chang
Treron phoenicopterus	3	Chang

*Codes: 1-3 are habitat specialists (forest-dependent to woodland birds), 4 denotes plantation or open woodland species, 5 common or cosmopolitan species. Sources: Anand *et al.* 2008, Beukema *et al.* 2007, Chang *et al.* 2017 (compiled from del Hoyo *et al.* 2016, LePage 2016).

Birds and Beans: Replication Code

Charlotte H. Chang & Krithi K. Karanth

10/25/2017

Description

This document contains replication code and a dataset to accompany the manuscript "Birds and beans: Comparing avian richness and endemism in *arabica* and *robusta* plantations in India's Western Ghats" by Charlotte H. Chang, Krithi K. Karanth, and Paul Robbins. An Rmarkdown HTML version of this code is posted at `http://rpubs.com/chwchang/WCS-India-Coffee` which may be more convenient for running analyses.

The central question addressed by this dataset is whether or not habitat specialist birds (forest-dependent, endemic, and IUCN Red-Listed species) exhibit different responses to *Coffea arabica* and *Coffea canephora* (*robusta* strain) plantations. Regional and global trends indicate that *robusta* production may be increasing at the cost of *arabica*, and this change may have biodiversity consequences as *robusta* is typically grown under full-sun, open habitat management, while *arabica* is more often shade-grown.

In this analysis, one can replicate habitat specialist individual-based rarefaction, asymptotic richness estimates, ordination, and distance-sampling abundance modeling.

Diversity analyses

Below, we load the Rdata containing the replication datasets. A description of each vector or dataframe is below:

- `FD.anon`: a vector of the column IDs for which species are forest-dependent
- `ED.anon`: a vector of column IDs for endemic species to India
- `IUCNbirds`: a vector of column IDs for IUCN Red-Listed species
- `coffee.SR.anon`: a data frame with counts for bird species across multiple estates with multiple replicates
- `FD.ds`: an R object from package `Distance` for forest-dependent bird abundance
- `End.ds`: Endemic abundance from package `Distance`
- `IUCN.ds`: IUCN Red-Listed species abundance from package `Distance`

```
###=====================================================
### Load diversity data
###=====================================================
## File download from GitHub repository - uncomment (remove leading hash) to run these two commands (whi
# githubURL <- "https://raw.github.com/charlottehchang/WCS-India-Coffee/master/WCS_Archiving.Rdata"
# download.file(githubURL,"WCS_Archiving.Rdata")

load("WCS_Archiving.Rdata") # make sure the path to the downloaded Rdata archive is correct--currently
ls() # This shows you what is now contained in the workspace
```

```
## [1] "coffee.SR.anon" "ED.anon"        "End.ds"         "FD.anon"
## [5] "FD.ds"          "IUCN.anon"      "IUCN.ds"
```

```
###=====================================================
### Estimate asymptotic richness
###=====================================================
# If you do not have this package, please first use the command:
```

```r
# install.packages(vegan) # Remove the leading hash to run this command
library('vegan') # load the vegan package and its functions
```

```
## Loading required package: permute

## Loading required package: lattice

## This is vegan 2.4-1
```

```r
      # Forest dependent analysis
print("Forest dependent species richness in arabica and robusta:")
```

```
## [1] "Forest dependent species richness in arabica and robusta:"
```

```r
pool <- specpool(coffee.SR.anon[,FD.anon],coffee.SR.anon$Crop)
pool
```

```
##         Species     chao    chao.se    jack1 jack1.se    jack2     boot
## Arabica      74 88.00509 10.3463570 86.92778 3.852901 93.88315 79.74846
## Robusta      66 66.33154  0.7547849 67.98925 1.406610 64.06434 67.79705
##          boot.se   n
## Arabica 2.132692 180
## Robusta 1.173085 186
```

```r
## IUCN Red-listed species richness
print("IUCN Red Listed richness in arabica and robusta:")
```

```
## [1] "IUCN Red Listed richness in arabica and robusta:"
```

```r
pool2 <- specpool(coffee.SR.anon[,IUCN.anon],coffee.SR.anon$Crop)
pool2
```

```
##         Species chao chao.se jack1 jack1.se jack2 boot       boot.se   n
## Arabica       3    3       0     3        0     3    3  1.311930e-57 180
## Robusta       3    3       0     3        0     3    3 1.991371e-111 186
```

```r
## Endemics
print("Endemic richness in arabica and robusta:")
```

```
## [1] "Endemic richness in arabica and robusta:"
```

```r
pool3 <- specpool(coffee.SR.anon[,ED.anon],coffee.SR.anon$Crop)
pool3
```

```
##         Species     chao  chao.se    jack1 jack1.se    jack2     boot
## Arabica      14 20.21528 7.512247 18.97222 2.223645 21.94994 16.10231
## Robusta      11 11.00000 0.000000 11.00000 0.000000  9.03220 11.30508
##           boot.se   n
## Arabica 1.1690611 180
## Robusta 0.5243744 186
```

```r
###=================================================
# ECOLOGICAL ANALYSES: INDIVIDUAL BASED RAREFACTION
###=================================================
  # Run commands of the following variety below:
Rare.FD <- specaccum(coffee.SR.anon[coffee.SR.anon$Crop=="Arabica",FD.anon],method="rarefaction")
plot(Rare.FD, xlab="Individual count",xvar="individuals",ylab="Rarefied richness")
```

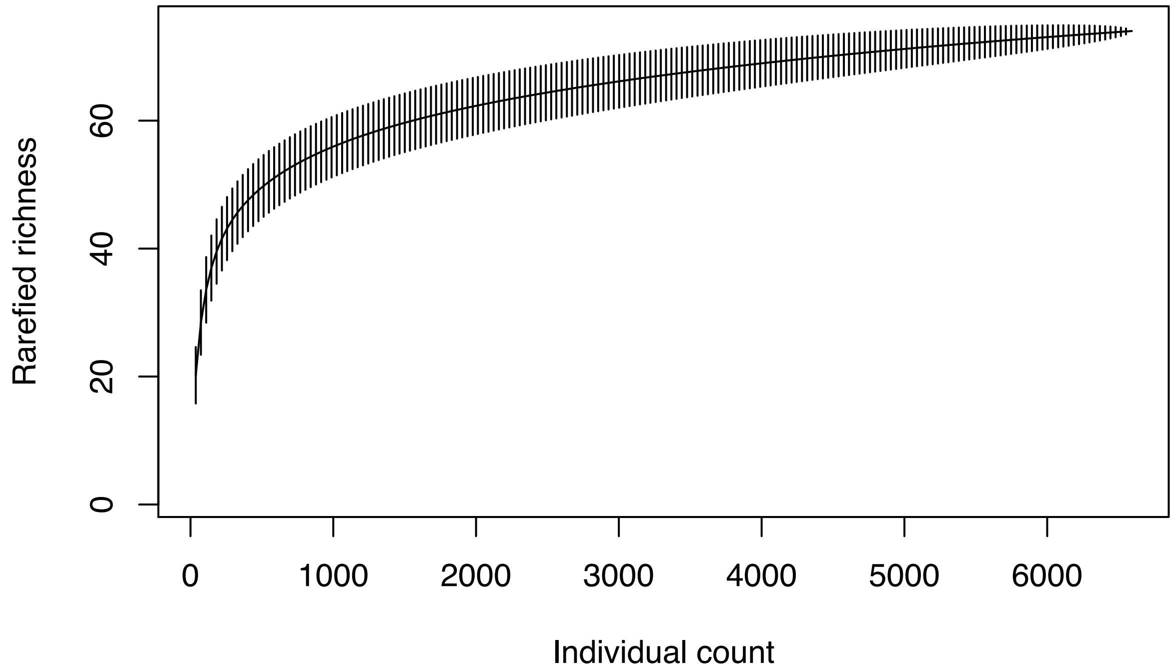


```
###=====================================================
# ECOLOGICAL ANALYSES: ORDINATION
###=====================================================

### Performing ordination
    # Repeat for ED.anon for endemics if so desired.
  # Calculate CCA
FD.CCA <- cca(coffee.SR.anon[,c(FD.anon)]~Crop, coffee.SR.anon)
  # Plotting example
plot(FD.CCA, type="points", xlim=c(-2,2), ylim=c(-2,2), display="species", scaling="species")
```

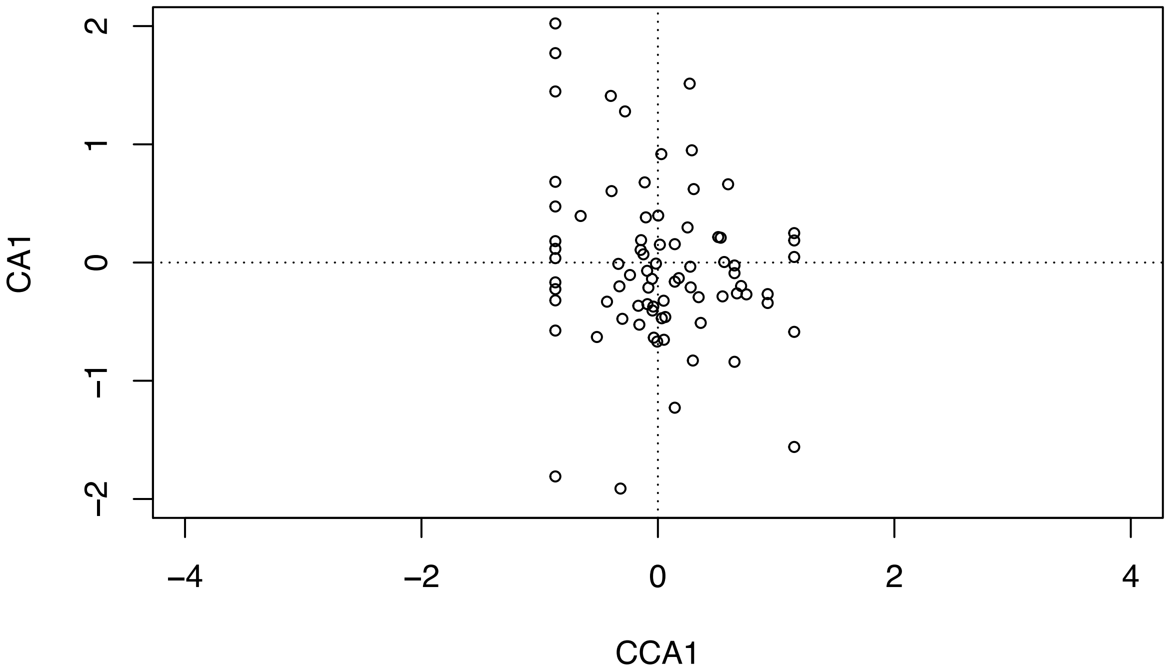

```
###=====================================================
# ECOLOGICAL ANALYSES: ABUNDANCE VIA DISTANCE
###=====================================================
ha.m2 <- 10000 # converting hectares to meters-square
    ## Obtaining statistics from Distance analysis (package 'Distance' in R)
print("Forest dependent individual average detection:")
```

```
## [1] "Forest dependent individual average detection:"
```

```
FD.ds$dht$individuals$average.p
```

```
## [1] 0.0933786
```

```
print("Forest dependent cluster average detection:")
```

```
## [1] "Forest dependent cluster average detection:"
```

```
FD.ds$dht$clusters$average.p
```

```
## [1] 0.1399554
```

```
    ## Obtaining individual bird densities
print("Range of FD individual densities across arabica versus robusta estates:")
```

```
## [1] "Range of FD individual densities across arabica versus robusta estates:"
```

```
print("Arabica range:")
```

```
## [1] "Arabica range:"
```

```
round(range(FD.ds$dht$individuals$bysample$Dhat[FD.ds$dht$individuals$bysample$Region=="Arabica"]*ha.m2
```

```
## [1]  0.38 48.69
```

```
print("Robusta range:")
```

```
## [1] "Robusta range:"
```

```
round(range(FD.ds$dht$individuals$bysample$Dhat[FD.ds$dht$individuals$bysample$Region=="Robusta"]*ha.m2
```

```
## [1]  0.38 28.27
```

```
    ## Obtaining flock size
# Cluster sizes
print("Forest dependent cluster sizes")
```

```
## [1] "Forest dependent cluster sizes"
```

```
FD.ds$dht$Expected.S
```

```
##    Region Expected.S se.Expected.S
## 1 Arabica   1.535362    0.04194179
## 2 Robusta   1.457918    0.03300256
## 3   Total   1.498419    0.02714972
```
